# Factors Influencing the Production of Extracellular Polysaccharides by the Green Algae *Dictyosphaerium chlorelloides* and Their Isolation, Purification, and Composition

**DOI:** 10.3390/microorganisms10071473

**Published:** 2022-07-21

**Authors:** Olga Kronusová, Petr Kaštánek, Görkem Koyun, František Kaštánek, Tomáš Brányik

**Affiliations:** 1 Department of Biotechnology, University of Chemistry and Technology Prague, Technická 5, 166 28 Prague, Czech Republic; kastanek@ecofuel.cz (P.K.); gorkem.koyun@vscht.cz (G.K.); tomas.branyik@vscht.cz (T.B.); 2 EcoFuel Laboratories s.r.o., Ocelářská 9, 190 00 Prague, Czech Republic; 3 Institute of Chemical Process Fundamentals of the CAS, Rozvojova 2/135, 165 02 Prague, Czech Republic; kastanek@icpf.cas.cz

**Keywords:** *Dictyosphaerium chlorelloides*, extracellular polysaccharides, photobioreactor

## Abstract

The freshwater green microalgae, *Dictyosphaerium chlorelloides* (CCALA 330), has the ability to produce extracellular polysaccharides (EPS). Conditions for optimum growth and EPS overproduction were determined in laboratory-scale tubular photobioreactors (PBR) with a working volume of 300 mL. Multiple limitations in nutrient supply were proven to be an effective method for EPS overproduction. Salinity stress was also applied to the culture, but no significant increase in EPS production was observed. The effects of different nitrogen sources were examined and the microalgae exhibited the fastest growth and EPS production in medium containing ammonium nitrate. Under determined optimal conditions, EPS concentration reached 10 g/L (71% of the total biomass) and a total biomass of 14 g/L at the end of 17 days cultivation. Pilot-scale cultivation was also carried out in a column type airlift photobioreactor (PBR) with a working volume of 60 L. A new and efficient methodology was developed for separating cells from the EPS-containing culture broth. Due to the strong attachment between cells and EPS, high-pressure homogenization was carried out before a centrifugation process. The EPS in the supernatant was subsequently purified using ultrafiltration. The green microalgae *Dictyosphaerium chlorelloides* may therefore be appropriate for the commercial production of EPS.

## 1. Introduction

One of the most valuable groups of compounds produced by microalgae are polysaccharides, whose various benefits are in medicine, dermo-cosmetics, nutrition, and various other applications [1,2,3,4,5]. Microalgae can use polysaccharides as protection against drying out, mechanical damage caused by sand, and as specific binding sites for viruses that protect the algal cell against infection [6,7,8,9]. The production of these substances is species-dependent, and the accumulation in microalgal cells or secretion to the culture medium are generally regulated by environmental stress response mechanisms that evolved for sensing and acclimatizing to changes in the local environment [10,11,12,13,14]. The major factors affecting microalgal polysaccharide production are particularly light, temperature, salinity, and nutrient composition [15,16,17,18]. The complex structures of polysaccharides and strong binding between polysaccharides in the culture medium and microalgal cells complicate the separation of these valuable compounds from the culture medium [19]. Extracellular polysaccharides (EPS) have a very variable composition and can be composed of homogeneous polysaccharides, such as glucans or galactans [20], or heteropolysaccharides, such as arabinogalactans, arabinoxylans, or glucomannans [21,22,23,24].

The similarity of the physicochemical properties of microalgal polysaccharides to other polysaccharides currently used in industry as gelling agents, stabilizers, emulsifiers and moisturizers makes microalgal polysaccharides a valuable alternative to existing industrial polysaccharides. One of their unique advantages over those of other polysaccharides, especially for commercial applications, is their product stability over a wide range of temperature, pH, light, and salinity [25]. Microalgal polysaccharides offer high stability of quality; they can be produced throughout the year under controlled conditions in the photobioreactor, and so are not affected by climatic variations, such as those polysaccharides traditionally extracted from plant material or macroalgae, which have more complex life cycles [26].

*Dictyosphaerium chlorelloides* is a freshwater green microalgae belonging to *Chlorophyceae*, with a worldwide distribution. A neutral pH medium for freshwater microalgae is generally used for its cultivation [27]. 

It is well known that downstream processing and extraction of microalgal EPS covers approximately 30–40% of its total production costs, therefore the effectiveness of separation technology plays a crucial role in the commercial production of algal EPS [28]. Some common methods of harvesting, such as flocculation, centrifugation, and sedimentation cannot be effectively exploited here due to the tight binding of EPS to the microalgae cell wall [29,30]. 

The most common method used for the extraction of algal polysaccharides is hot water extraction [31]. More advanced extraction techniques, such as ultrasonic-assisted extraction, supercritical extraction, and membrane separation were also developed [32,33,34,35]. Additionally, both water and enzymatic extraction can be applied to obtain polysaccharides [36]. Some studies also considered using precipitation techniques [37,38]. Patel et al. compared the efficiency of filtration and solvent precipitation techniques (using methanol, ethanol, and isopropanol) in separating EPS from *Porphyridium* sp. It was concluded that a tangential flow filtration system was a promising technology for the selective collection and desalting of these polymers [39]. Some publications discuss the separation of EPS in combination, or separately, with ultrasonication and high-pressure homogenization, which can be effective for separating bio-molecules from microalgae [40,41]. To avoid using solvents and methods that cannot be applied at an industrial scale, alternative techniques for separating valuable components must be sought. One such candidate is ultrafiltration, which can be scaled up to an industrial level. A few studies investigated this technique on microalgae to purify polysaccharides, specifically polysaccharides of *Porphyridium cruentum* [39], *Spirulina platensis*, and *Chlorella pyrenoidosa* [42].

This paper aims to identify the optimum conditions to maximize production of EPS produced by *Dictyosphaerium chlorelloides* in laboratory 300 mL photobioreactors by applying various stress factors and optimizing EPS harvesting.

## 2. Materials and Methods

### 2.1. The Strain 

Microalga *Dictyosphaerium chlorelloides* CCALA 330 was obtained from the Culture Collection of Autotrophic Organisms (strain CCALA 330, habitat: fish pond Trebon, Czech Republic) at the Botanical Institute of the Academy of Sciences, Czech Republic.

### 2.2. Chemicals 

All chemicals used were in *pro analysis* quality, origin: Sigma Aldrich and/or Lachema, CR, Penta, CR. Anthrone solution: 400 mg anthrone dissolved in 200 mL of 72% H_2_SO_4_. Glucose solution: 100 mg of glucose was dissolved in100 mL of 30% HClO_4_.

### 2.3. Instruments 

Centrifuges: Eppendorf centrifuge 5424, DE; Jouan C312, US; Wise Spin, DE. High-pressure homogenizer: IKA labor pilot 2000/R, DE. Spectrophotometer: Spekol 11, Carl Zeiss, DE. Ultrafiltration: Pellicon 2 mini, Millipore, DE. Viscometer: DV-1, Brookfield, US. pH meter: MultiCalpH256, WTW, CR. Vortex mini shaker: IKA Genius3, DE. Illuminative LED panel: Briklis, CR. Column 300 mL photobioreactor and Airlift 60 L photobioreactor: Briklis, CR. GC-FID Shimadzu GC 2010 Shimadzu, Japan. Bruker AVANCE III- Bruker NMR Technology 125.7 MHz for 13C/HSQC, USA.

### 2.4. Vertical Column Photobioreactor (300 mL)

The inner diameter of the photobioreactor was 36 mm, the height of the photobioreactor was 50 cm. Air enriched with 2% CO_2_ (*v*/*v*) was introduced at a flow rate of 10 L h^−1^ per tube. The temperature of 25 °C was previously selected as optimal for maximum EPS production. The cultures were continuously illuminated by a LED light panel at an irradiance level of 230 µE/m^2^/s. The pH was kept within the range of 6.5–7.5 using solutions of 0.1M KOH and 0.1M HCl. The culture was aerated and stirred by sterilized air enriched with 2% CO_2_. For inoculation, cultures were grown in a basic full medium. The inoculum was cultivated for 7 days. The medium (270 mL) was inoculated with 30 mL of cell suspension (X_T_ 6 g/L after 7 days of cultivation in the exponential growth phase).

### 2.5. Airlift Column Photobioreactor (60 L)

The inner diameter of the photobioreactor was 292 mm, the height of the photobioreactor was 100 cm. The photobioreactor glass wall thickness was 21 mm. Air enriched with 2% CO_2_ (*v*/*v*) was introduced at a flow rate of 80 L h^−1^. The photobioreactor was illuminated by four LED light panels at an irradiance level of 250 µE/m^2^/s. The cultivation temperature was 25 °C.

### 2.6. Growth Medium 

Full medium contained the following macronutrients in mg/L: NaNO_3_ (1558); KH_2_PO_4_ (237); MgSO_4_.7 H_2_O (204); C_10_H_12_O_8_N_2_NaFe (40); CaCl_2_ (88) and micronutrients in mg/L: H_3_BO_3_ (0.83); CuSO_4_.5 H_2_O (0.95); MnCl_2_.4 H_2_O (3.29); CoSO_4_.7 H_2_O (0.62); ZnSO_4_.7 H_2_O (2.68); (NH_4_)_6_Mo_7_O_24_.4H_2_O (0.17); and (NH_4_)VO_3_ (0.01). Different nitrogen sources alongside sodium nitrate (NaNO_3_), such as ammonium sulphate (NH_4_SO_4_), ammonium nitrate (NH_4_NO_3_), and urea ((NH_2_)_2_CO), were also applied using a fixed nitrogen concentration. To determine the appropriate degree of dilution for maximum production of EPS, all nutrients in the full medium were diluted 3, 6, and 12-fold [43].

### 2.7. Biomass Cell Concentration Measurement

The algal cells dry weight (Xc) was determined using a calibration curve (A_750_ vs. Xc) obtained from algal culture harvested on the 3rd day of cultivation without EPS in the culture medium. The absorbance (A_750_) was determined in the range of 0.1–0.8 [44]. Dry weight was determined using algal culture (without EPS) after centrifugation at 3000 rpm for 10 min and drying at 105 °C in glass tubes, for at least eight hours, to a constant weight. For the determination of dry matter achieved in high-salinity cultivations, salts in a 2 mL medium were washed on filter paper with 10 mL of distilled water. Subsequently, the filter paper with the biomass was dried at 70 °C, for at least 12 h, to a constant weight [45,46]. Total algal biomass dry weight (X_T_) was determined as the sum of algal cells dry weight (X_C_) and algal EPS dry weight (X_EPS_).

### 2.8. Quantification of Extracellular Polysaccharides

The method was based on a slightly modified protocol for the determination of starch accumulated in microalgae *Chlorella* sp. [47]. The determination of starch in this protocol is based on the reaction of monosaccharides after hydrolysis with an anthrone reagent. This reaction gives a blue color, which is measured at 625 nm in concentrated sulfuric acid.

Isolation of EPS: 1 mL total volume of glass beads (Zirconium, no. 8) was added to 2 mL of the microalgal culture in tubes and the mixture was vortexed for 15 s at level 6 (maximum setting) of the vortex mini shaker. The tubes were centrifuged for 30 min at 3400 rpm. After centrifuging, the cells were separated and observed among glass beads in the sediment. The supernatant with EPS present was collected.

Hydrolysis of EPS to monosaccharides: 400 µL of liquid were transferred to glass tubes and 6 mL of 30% HClO_4_ were added to each tube. Hydrolysis of the samples was carried out at room temperature for 45 min and homogenized by vortexing every 10 min.

The color reaction of monosaccharides with anthrone: 0.5 mL samples from each tube were put into glass vials in an ice bath. Next, 2.5 mL of anthrone solution was pipetted into the tubes. All samples were then placed into a boiling water bath for 8 min, followed by being placed immediately into an ice bath to cool to room temperature (20 °C). Samples were mixed well with a vortex mixer. Absorbance was measured at 625 nm in glass cuvettes (width 1 cm) against a blank (30% HClO_4_).

Evaluation of the concentration of EPS (X_EPS_): The dry weight of EPS produced was obtained using a calibration based on the known mass of glucose after a reaction with the anthrone reagent. The data obtained were compared with the absorbance data obtained from the results of the reaction of the hydrolysate of EPS.

### 2.9. Viscosity Measurement

During cultivation, the viscosity of the culture medium increased due to the production of EPS. Therefore, the viscosity of the medium was measured periodically, and the viscosity data were calibrated with the EPS content, as estimated by the anthrone method. All viscosity data were collected using a Brookfield viscometer DV-I (spindle 2) at a shear rate of 100 rpm. Only the values between torque ranges of 10% and 90% were considered. 

### 2.10. Staining of Extracellular Polysaccharides

The staining procedure was inspired by the Alcian blue staining protocol [48]. The solution for staining EPS was prepared as follows: 0.1 g of powder reagent with Alcian blue was added to 9.7 mL of distilled water. After stirring this solution, 300 mL of concentrated acetic acid was added to it. The mixture was then filtered through filter paper into a glass tube. The test algal culture was placed onto a glass slide and fixed above a flame. The microalgal culture was stained with the prepared solution of Alcian blue for 15 min. The slide was then washed under flowing water for 5 min and observed under the microscope. 

### 2.11. Salinity Stress

A high-salinity environment was created by adding 1.5, 3, 6, 12, and 24 g/L NaCl to the selected growth medium (6-fold diluted full medium).

### 2.12. Separation of Cells from the Culture Medium

High-pressure homogenization and centrifugation were sequentially used to separate EPS from microalgae. The homogenization process was performed with an IKA Labor Pilot 2000/4 high-pressure homogenizer under pressures of 250, 500, 1000, 1500, and 2000 bars, and a speed of 6 L h^−1^. Due to its highly viscous nature, the medium was 2× diluted with distilled water before homogenization. Afterward, centrifugation was carried out at 3400 rpm for 10 min. Absorbance (at 750 nm) and EPS concentrations in supernatants were measured. Additionally, the supernatant was visualized under the microscope to check the efficiency of the separation of the EPS.

### 2.13. Purification of Extracellular Polysaccharides

The process of ultrafiltration was also applied using the tangential flow filtration pilot unit Millipore, Pellicon 2 mini for the desalination culture medium and concentration of the EPS. Membranes had a normal molecular weight cut-off (NMWCO) of 5 kDa, with a surface of 0.1 m^2^. Firstly, the supernatant was 2× diluted with distilled water, and then pumped onto the membrane. The permeate fraction was collected and the retentate fraction was returned to the feeding tank. At the end of the experiment, EPS concentrations were measured by the anthrone method in the retentate and permeate fractions for mass balance.

### 2.14. Determination of Extracellular Polysaccharides Composition

Polysaccharides were extracted from the algal biomass using DMSO. Subsequently, the proteins were removed using proteinases (pepsin). Monosaccharides were determined after acid hydrolysis by gas chromatography [49]. The relative molecular weight was determined by gel permeation chromatography. The purified fractions were subsequently analyzed by nuclear magnetic resonance NMR a 13C APT NMR (program MetReNova 10.0 COSY, HSQC/HMQC, and HMBC) [49].

### 2.15. Statistical Analysis

Where applicable the results are presented as average values with standard deviations. The experimental data were statistically evaluated using one-way ANOVA (analysis of variance) with post hoc Tukey HSD test. All comparative and quantitative statements were based on a probability of *p* < 0.05. Statistical analyses were performed using Statistica 12.0 (StatSoft Inc., Tulsa, OK, USA).

## 3. Results

### 3.1. Laboratory Scale 300 mL Column Photobioreactors—Optimization of Cultivation Medium for Algal EPS Production

#### 3.1.1. Nutrient Limitation

**Figure 1:**
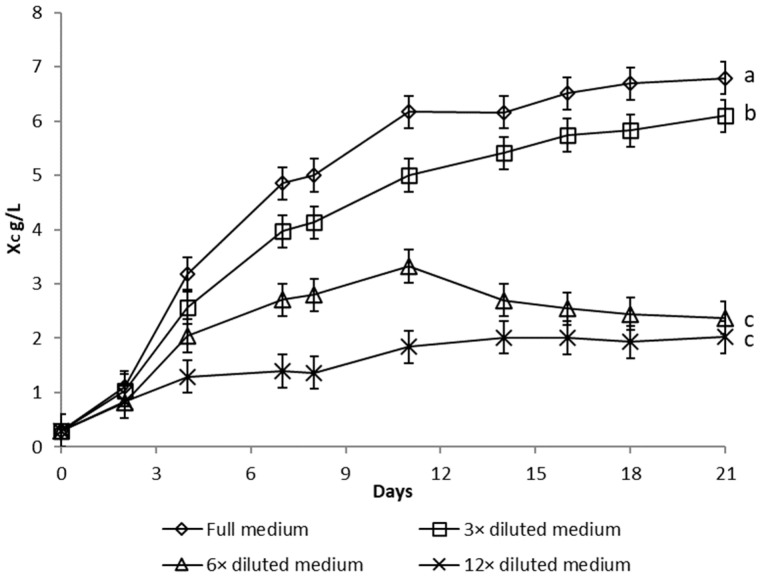
Microalgal cells dry weight (X_C_) of *Dictyosphaerium chlorelloides* in full and nutrients limited media. Means with at least one letter the same are not significantly different (*p* > 0.05). Comparison of statistical significance is presented for X_C_ on the 21st day of cultivation. Error bars indicate the standard deviations. All experiments were carried out in three biological replicates.

**Figure 2:**
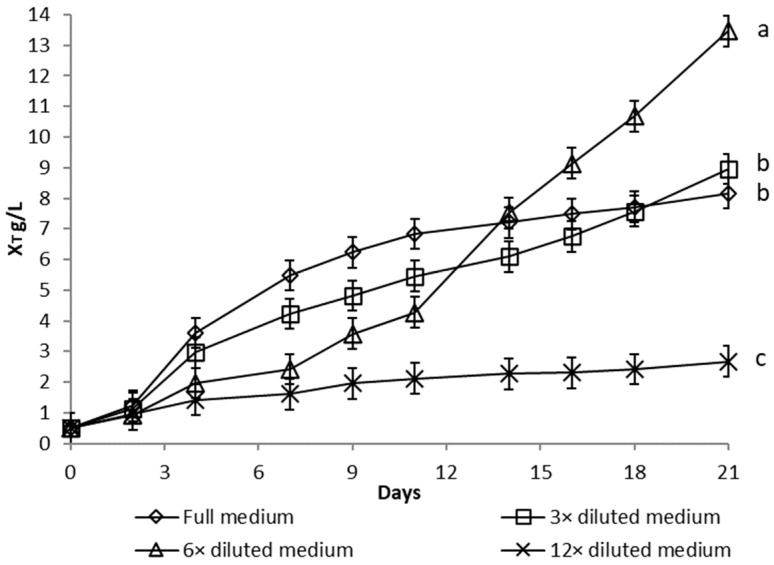
Total biomass dry weight (X_T_) of *Dictyosphaerium chlorelloides* (cells and extracellular polysaccharides) in full and nutrients limited media. Means with at least one letter the same are not significantly different (*p* > 0.05). Comparison of statistical significance is presented for X_T_ on the 21st day of cultivation. Error bars indicate the standard deviations. All experiments were carried out in three biological replicates.

**Figure 3:**
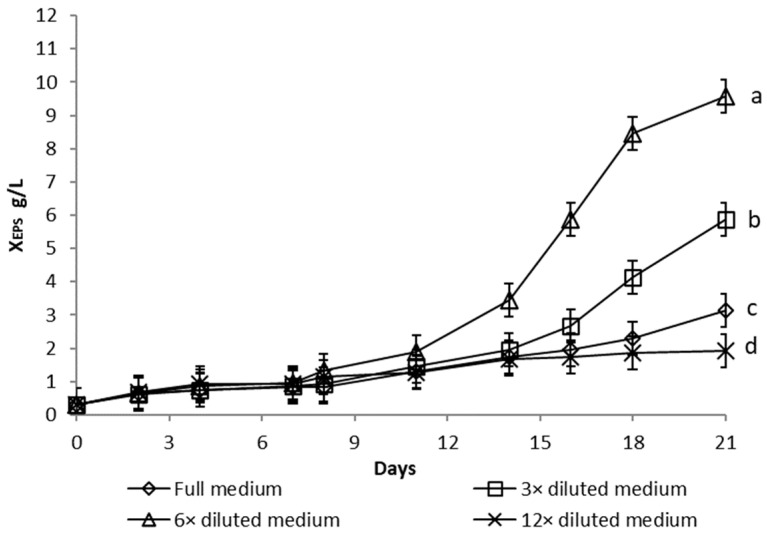
The dry weight of extracellular polysaccharides (X_EPS_) produced during cultivation of *Dictyosphaerium chlorelloides* in full and nutrients limited media. Means with at least one letter the same are not significantly different (*p* > 0.05). Comparison of statistical significance is presented for X_EPS_ on the 21st day of cultivation. Error bars indicate the standard deviations. All experiments were carried out in three biological replicates.

Growth curves of *Dictyosphaerium chlorelloides* in media, with varying nutrient concentrations, are described as functions of microalgal cells dry weight (Figure 1), total biomass dry weight (Figure 2, sum of microalgae cells and EPS dry weight), and the EPS dry weight produced (Figure 3).

Significant growth of *Dictyosphaerium chlorelloides* cell dry weight (X_C_) was detected in full medium (Figure 1). Conversely, dilution of the full medium led to a decrease in microalgal cell formation, as shown in the growth curves (Figure 1). The final Xc in full medium was significantly higher than in a 3-fold diluted medium (*p* = 0.032). However, total biomass dry weight (X_T_), which includes both microalgal cells and EPS, increased progressively in a culture in a 6-fold diluted full medium (Figure 2). Likewise, the highest overproduction of EPS (X_EPS_) was achieved in a culture with a 6-fold diluted full medium (Figure 3). Both final X_T_ and X_EPS_ in a 6-fold diluted full medium were the highest with a statistically significant difference (*p* ˂ 0.05). The onset of EPS formation was already observable from the 8th day of cultivation. The concentration of EPS was 10 g/L at the end of 17 days (Figure 3), which represents up to 71% of X_T_. The second most pronounced formation of EPS was observed in the case of a 3-fold diluted full medium, where the EPS represented 66% of X_T_. 

#### 3.1.2. Salinity Stress

**Figure 4:**
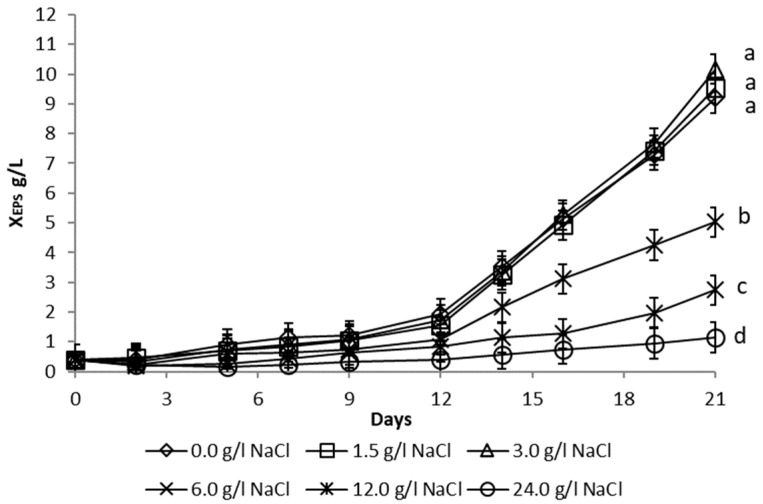
Influence of culture medium salinity on the production of extracellular polysaccharides (X_EPS_) during cultivation of *Dictyosphaerium chlorelloides* in a 6-fold diluted full medium. Means with at least one letter the same are not significantly different (*p* > 0.05). Comparison of statistical significance is presented for X_EPS_ on the 21st day of cultivation. Error bars indicate the standard deviations. All experiments were carried out in three biological replicates.

These experiments were carried out with a 6-fold diluted full medium, which previously demonstrated to be the most efficient for EPS production. The growth of microalgae and EPS formation (Figure 4) was, however, gradually inhibited in medium containing more than 6 g/L NaCl. In a 6-fold diluted full medium with an addition of 0 to 3 g/L NaCl, microalgae showed similar biomass growth and the final X_EPS_ formation showed a statistically not significant (*p* > 0.05) difference (Figure 4).

#### 3.1.3. Nitrogen Source

Microalgae may exhibit different growth patterns with different nitrogen sources due to differences in their ability to utilize these sources. The effects of four different nitrogen sources—sodium nitrate (NaNO_3_), ammonium sulphate (NH_4_SO_4_), ammonium nitrate (NH_4_NO_3_), and urea ((NH_2_)_2_CO)—on algal cell growth and EPS production were compared. A 6-fold diluted full growth medium was used in all cases, with only the nitrogen source being changed. The molar concentration of nitrogen and all other environmental conditions were constant. 

**Figure 5:**
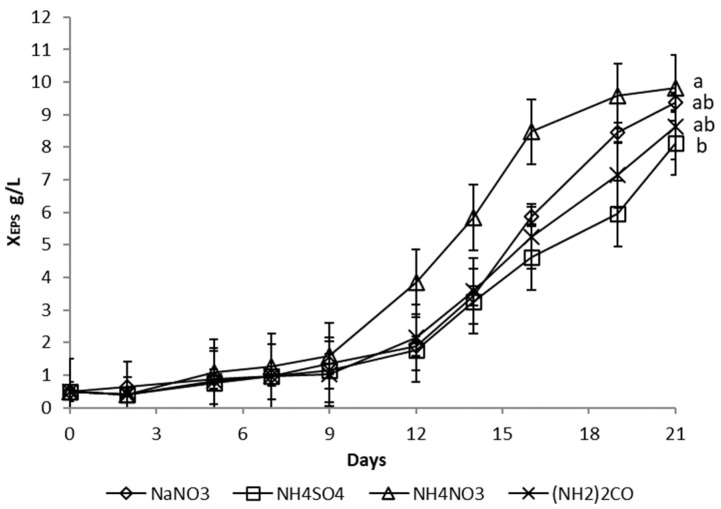
Influence of a nitrogen source on the production of extracellular polysaccharides (X_EPS_) during cultivation of *Dictyosphaerium chlorelloides* in a 6-fold diluted full medium. Means with at least one letter the same are not significantly different (*p* > 0.05). Comparison of statistical significance is presented for X_EPS_ on the 21st day of cultivation. Error bars indicate the standard deviations. All experiments were carried out in three biological replicates.

Algae showed the highest rate of initial biomass growth in the cultivation medium containing NH_4_NO_3_, followed by NaNO_3_, (NH_2_)_2_CO, and finally NH_4_SO_4_. The same applied to EPS production, which started on the 9th day in the NH_4_NO_3_ medium, whereas it occurred on the 12th day in a medium containing all other nitrogen sources (Figure 5). However, the final X_EPS_ in a 6-fold diluted medium with NH_4_NO_3_, NaNO_3_, and (NH_2_)_2_CO were similar, with statistically not significant difference (*p* > 0.05).

### 3.2. Algal Cultivation in 60 L Photobioreactor

**Figure 6:**
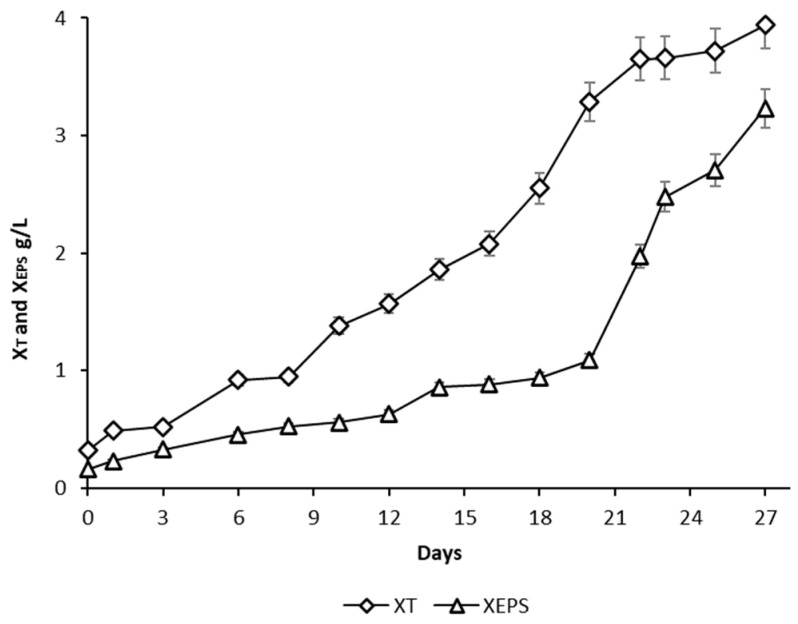
Total biomass dry weight (X_T_) and the extracellular polysaccharides dry weight (X_EPS_) produced during cultivation of *Dictyosphaerium chlorelloides* in a 60 L airlift photobioreactor in a 6-fold diluted full medium. Error bars indicate the standard deviations. The experiment was carried out in one biological replicate.

For the production of EPS by microalgae *Dictyosphaerium chlorelloides* in a 60 L airlift photobioreactor, cultivation conditions were used according to previous experiments in 300 mL column photobioreactors. A nutrient limitation strategy with a 6-fold diluted medium was used. The final X_T_ and X_EPS_ values were 4.0 and 3.2 g/L, respectively (Figure 6). Compared to column photobioreactors (Figure 2 and Figure 3), the maximum X_T_ and X_EPS_ values were lower, but the percentage amount of EPS in the X_T_ was high (X_EPS_ 71% and 80%, respectively).

### 3.3. Separation of EPS from Algae Cells

**Figure 7:**
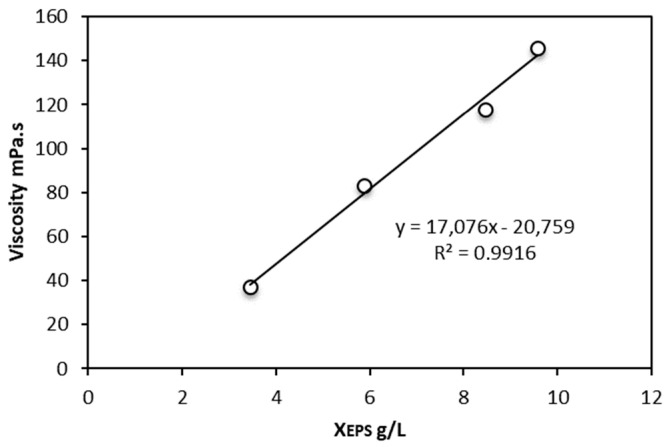
Microalgal culture viscosity is affected by the concentration of extracellular polysaccharides (X_EPS_) in the culture medium.

The dynamic viscosity of the culture medium was collected from different cultivations and correlated with X_EPS_ (Figure 7). However, algal EPS is tightly bound to the algal cell wall and it is not possible to separate them by centrifugation without high-pressure homogenization (Table 1). The culture medium used for the homogenization experiments was obtained after cultivation of microalgae in an airlift photobioreactor. Before introducing the culture broth to the homogenizer, it was diluted 2-fold due to its high viscosity. The amount of EPS in the feed before entering the homogenization process was therefore calculated as 1.6 g/L. 

The attachment between microalgal cells and EPS was relaxed using high-pressure homogenization. Subsequently, the cells were separated easily from the culture medium by centrifugation. The optimum pressure for homogenization was determined to be 1000 bars, which resulted in the highest X_EPS_ in the supernatant. At higher pressures, chlorophyll was visualized in the supernatant due to the gradual increase in algal cell wall destruction and the release of cell contents into the medium. This caused the Xc to rise again at homogenization pressures above 1000 bar. On the other hand, at homogenization pressures below 1000 bar, EPS (X_EPS_) and cells (Xc) separated less efficiently during the subsequent centrifugation (Table 1). 

### 3.4. Purification of EPS

The supernatant from the homogenization process was diluted 2-fold before entering ultrafiltration to prevent any possible damage to the membrane. Accordingly, the X_EPS_ and volume of the feed were determined as 0.68 g/L in 8.4 L, respectively. The retentate was recycled until its volume dropped to approximately 16% of the initial injected volume.

The X_EPS_ in the isolated retentate was determined to be 3.39 g/L (Table 2). During the ultrafiltration process, 83% of the initial EPS amount was retained in the retentate fraction, 9% was found in the permeate, and 8% was lost attached to the membrane.

### 3.5. Determination of EPS Composition

According to the procedure specified in the methodology, the monosaccharide composition of EPS from *Dictyosphaerium chlorelloides* CCALA 330 was determined. The monosaccharide composition of EPS was (in wt.%): galactose (48.8), rhamnose (25.2), mannose (20.8), xylose (2.7), glucose (2.2), and arabinose (0.3).

## 4. Discussion

**Figure 8:**
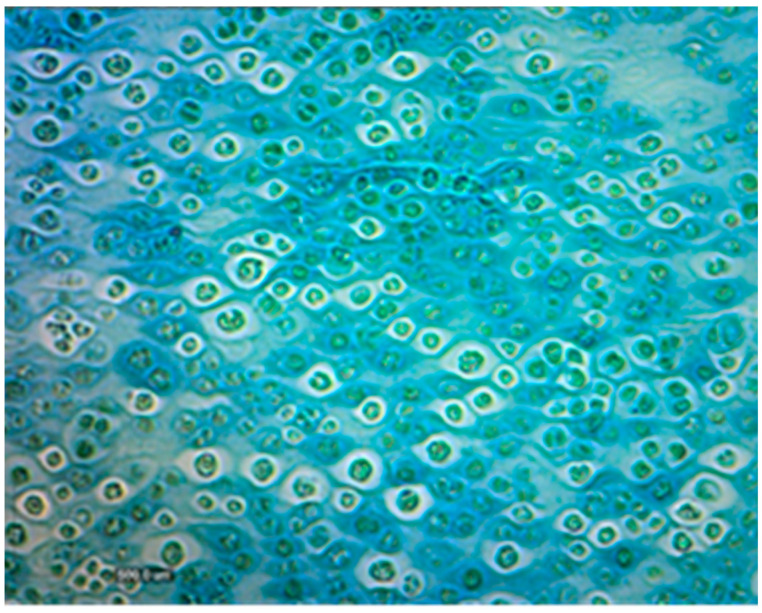
*Dictyosphaerium chlorelloides* cells stained with Alcian blue in a 6-fold diluted medium. The EPS are visualized in blue color as capsules of EPS around the cells (4000×).

The 6-fold diluted full medium induced significant EPS production (70% increase), in *Dictyosphaerium chlorelloides* culture, due to optimal nutrient limitation [15,43,47]. In the harvested culture medium (X_T_ 14 g/L), 71% of total biomass was represented by EPS (X_EPS_ 10 g/L) and 29% by microalgal cells (X_C_ 4 g/L). As a result of staining with Alcian blue, it was possible to observe a culture medium where microalgal cells produce a visible polysaccharide capsule around cells (Figure 8).

The 6-fold diluted medium of all nutrients proved to be a more effective induction of stress and EPS overproduction than the limitation of individual nutrients of the medium (which was later also tested but appeared to be ineffective). At the same time, a medium with more than 6-fold diluted nutrients probably does not provide enough nutrients for EPS synthesis, and the productivity of EPS decreases. Similar success was achieved with the microalga *Parachlorella kessleri*, where a 5–10 diluted medium led to lipid overproduction. The authors suggest the two-phase cultivation strategy for lipid overproduction consists of the fast growth of *P. kessleri* culture grown in the complete medium to produce sufficient biomass followed by the dilution of nutrient medium to stop growth and cell division by limitation of all elements, leading to induction of lipid production and accumulation up to 60% DW [43]. 

The maximum X_EPS_ (10 g/L) produced by *Dictyosphaerium chlorelloides* was significantly higher when compared to 1.32 g/L of sulphated polysaccharides observed for the marine microalgae *Porphyridium* sp. cultivated in a flat plate glass photobioreactor [50]. Under optimal conditions, *Dictyosphaerium chlorelloides* produced EPS with a productivity of 0.625 g/L/day. This is significantly higher than the EPS productivity of *Porphyridium marinum*, where the maximum X_EPS_ of 2.5 g/L in 26 days was produced with a productivity of 0.096 g/L/day [51].

At the genetic level, only the effect of nutrient nitrogen limitation on lipid accumulation in microalgae cells was investigated and described so far. Where nitrogen deprivation led to an increase in the expression of genes involved in fatty acid biosynthesis and a decrease in photosynthesis and lipid catabolism enzymes [52]. If there is higher than a 60% production of a product of secondary metabolism (lipids, polysaccharides), it is called hyperproduction. It is rare, but it can be achieved with appropriate cultivation conditions [43].

The main sugar composition of EPS from *Porphyriridium* sp. (xylose, galactose, and glucose) [37] was different from the EPS from *Dictyosphaerium chlorelloides*. At maximum X_EPS_ the culture medium with *Dictyosphaerium chlorelloides* reached a dynamic viscosity of 150 mPa.s, which is approximately 150 times higher than the viscosity of water at 20 °C.

Green seaweed *Ulva* sp. is also able to produce a high proportion of polysaccharides in biomass, up to 65% of dry weight [50]. This is close to the amount reached by *Dictyosphaerium chlorelloides* (71% of dry weight). However, when selecting the proper production taxon, various aspects have to be considered. One of them is the ease and productivity of cultivating microalgae in PBR compared to macroalgae.

Subsequently, the effect of medium salinity was examined. Growth and EPS formation were inhibited in medium containing NaCl above 6 g/L. The reason for this phenomenon could be damage to the photosynthetic apparatus in high-salinity environments, thereby causing a decrease in photosynthetic efficiency [53].

Another important process parameter in microalgal EPS production is the type of nitrogen source. Choosing the right nitrogen source has both physiological and economic implications. The highest initial growth rate and the earliest start of the stationary phase were observed when the culture medium contained NH_4_NO_3_. The fastest growth can be explained by the higher assimilation rate of NH_4_NO_3_ in *Dictyosphaerium chlorelloides* when compared to other nitrogen sources. Similarly, *Chlorella ellipsoidea* exhibited maximum growth rate in a medium containing NH_4_NO_3_ [54,55], whereas another species, *Chlorella pyrenoidosa*, grew better and produced the maximum amount of lipids in a medium containing NaNO_3_ [56]. In contrast, *Porphyridium* sp. produced the highest amount of EPS by assimilating KNO_3_ [57]. Therefore, the assimilation of different nitrogen sources by microalgae and their effect on growth and metabolite formation can be considered species-dependent.

For efficient separation of cells from the culture broth, a new methodology was developed to overcome the problem of the strong attachment between cells and EPS, which complicates the separation process. Bonds between cells and EPS were partially released using high-pressure homogenization. Subsequently, cells were separated easily from the culture medium by centrifugation. The optimum pressure for homogenization was determined at 1000 bars, which resulted in the best separation efficiency. Finally, EPS in the supernatant ware purified using ultrafiltration. The amount of 83% of the initial EPS was retained in the retentate fraction at the end of the ultrafiltration cycle.

High-pressure pressure homogenization and ultrafiltration, developed for the isolation of EPS from total microalgal biomass and subsequent concentration may be advantageous in the industrial production of EPS and for further commercial applications.

## 5. Conclusions

Currently, there are two main types of natural EPS on the market for skincare and hydration. The first is hyaluronic acid powder produced by the bacterium *Streptococcus zooepidemicus* [58]. The second product is Kelco-Care^®^ diutane gum, which is a water-soluble biopolymer produced fermentatively by the bacteria *Sphingomonas* [59]. These polysaccharides are used to produce natural skin serums, creams, and other emulsions because of increasing demand for natural cosmetics. *Dictyosphaerium chlorelloides* EPS, therefore, seem to be another interesting alternative for an active ingredient in cosmetics based on natural exopolysaccharides. Research into the antiviral activity of these EPS would also be of interest for the future, e.g., as algae *Porphyridium purpureum* EPS was shown to contain specific virus binding sites that protect the cell from infection. There are also reports on algal EPS antiviral activities against papillomaviruses and COVID-19 viruses [60,61]. Sulfated algal polysaccharides may also have pharmacological activities, such as antitumor, antimicrobial, antioxidant, anticoagulant, and anti-inflammatory effects [62]. Our work suggests that the freshwater microalgae *Dictyosphaerium chlorelloides* CCALA 330 is a very promising source for commercial EPS production and application. 

## Figures and Tables

**Table 1:** Microalgal cells dry weight (Xc) and extracellular polysaccharides dry weight (X_EPS_) in supernatants after homogenization under different pressures followed by centrifugation.

Pressure (Bars)	X_C_ (g/L)	X_EPS_ (g/L)
0	0.96	1.11
250	0.58	1.25
500	0.24	1.31
1000	0.07	1.50
1500	0.17	1.51
2000	0.20	1.51

**Table 2:** The dry weight of extracellular polysaccharides (X_EPS_) and volumes (V) of feed, retentate and permeate fractions in the ultrafiltration process.

Fraction	X_EPS_ (g/L)	V (L)
Feed	0.68	8.4
Retentate	3.39	1.4
Permeate	<0.01	7.0

## Data Availability

Detailed data can be provided upon request from the authors of the article.
